# Brain and liver pathology, amyloid deposition, and interferon responses among older HIV-positive patients in the late HAART era

**DOI:** 10.1186/s12879-017-2246-7

**Published:** 2017-02-17

**Authors:** Isaac H. Solomon, Umberto De Girolami, Sukrutha Chettimada, Vikas Misra, Elyse J. Singer, Dana Gabuzda

**Affiliations:** 1 Department of Pathology, Brigham and Women’s Hospital, Boston, USA; 2 Department of Cancer Immunology and Virology, Dana-Farber Cancer Institute, Boston, USA; 3 Department of Neurology and UCLA National Neurological AIDS Bank (NNAB), David Geffen School of Medicine, University of California at Los Angeles, Los Angeles, USA; 4 Department of Neurology, Harvard Medical School, Boston, USA; 5 CLS 1010, 450 Brookline Ave, Boston, MA 02215 USA

**Keywords:** HIV, HIV-associated neurocognitive disorders, Alzheimer’s disease, Amyloid, Neuropathology, Liver, White matter, Inflammation, Interferon response

## Abstract

**Background:**

HIV+ patients on highly active antiretroviral therapy (HAART) with suppressed viral loads have a low incidence of HIV-associated dementia, but increased prevalence of milder forms of HIV-associated neurocognitive disorders (HAND). These milder forms of HAND are often associated with minimal histological abnormalities, and their pathophysiology is unclear. Comorbidities, altered amyloid metabolism, accelerated brain aging, and activated interferon responses are suspected to play a role in HAND pathogenesis in HAART–treated persons.

**Methods:**

To investigate associations between liver disease, accelerated brain aging, and HAND in HIV+ patients in the late HAART era (2002–2015), we studied liver and brain autopsy tissues from 53 older subjects evaluated at UCLA and BWH using histopathological stains, a sensitive fluorescent amyloid stain (AmyloGlo), and targeted gene expression profiling (NanoString).

**Results:**

The majority of HIV+ subjects (median age 56) were on HAART (89.3%) with last pre-mortem plasma viral load <400 copies/mL (81.5%); 50% had CD4+ counts <200 cells/μL. Compared to HIV- controls (median age 65), HIV+ subjects had more cancer (*p* = 0.04), illicit drug use (*p* <0.00001), and HCV co-infection (*p* = 0.002), less cardiovascular disease (*p* = 0.03), and similar prevalence of cerebrovascular disease (~40%), hypertension, hyperlipidemia, and diabetes. Deep frontal white matter showed increased gliosis in HIV+ subjects vs. HIV- controls (*p* = 0.09), but no significant differences in myelin loss, blood vessel thickening, or inflammation. Liver showed more severe fibrosis/cirrhosis (*p* = 0.02) and less steatosis (*p* = 0.03) in HIV+ subjects, but no significant differences in inflammation, blood vessel thickness, or pigment deposition. There were no significant associations between liver and brain pathologies. AmyloGlo staining detected large amyloid deposits in only one HIV+ case (age 69 with Alzheimer’s disease pathology) and two HIV- controls (ages 66 and 74). White matter from HIV+ cases vs. HIV- seronegative controls showed a trend (*p* = 0.06) towards increased interferon response gene expression (ISG15, MX1, IFIT1, IFIT2, and IFITM1).

**Conclusions:**

Gliosis and cerebrovascular disease, but not accelerated amyloid deposition, are common brain pathologies among older HIV+ patients in the late HAART era. Although HIV+ subjects had more cirrhosis, liver pathology was not associated with any consistent pattern of brain pathology. Cerebrovascular disease, interferon responses, and neuroinflammation are likely factors contributing to brain aging and HAND in older HIV+ patients on current HAART regimens.

**Electronic supplementary material:**

The online version of this article (doi:10.1186/s12879-017-2246-7) contains supplementary material, which is available to authorized users.

## Background

HIV-associated dementia (HAD), the most severe form of HIV-associated neurocognitive disorders (HAND), affected approximately 20% of AIDS patients prior to death in the pre-highly active antiretroviral therapy (HAART) and early HAART eras (1981–2001) [1, 2]. In the pre-HAART era, HAD pathogenesis was largely attributed to viral replication in brain macrophages/microglia, immune activation in the CNS, and neuronal loss, due to the effects of neurotoxic viral proteins and pro-inflammatory cytokines. This model was consistent with the classic neuropathologic findings in HIV encephalitis (HIVE), characterized by multinucleated giant cells, diffuse microgliosis, microglial nodules, neuronal loss, astrocyte hypertrophy, myelin pallor, and immunoreactivity for HIV antigens including p24 [3, 4]. In the late HAART era (2002–present), milder forms of HAND, such as minor neurocognitive disorder (MND) and asymptomatic neurocognitive impairment (ANI), are more prevalent, with estimates ranging from 20 to 50% of HAART-treated HIV+ individuals depending upon the sample studied [2, 5, 6]. Brains from these individuals often show minimal or non-specific histopathologic changes regardless of their pre-mortem cognitive status [7, 8]. Consequently, the pathophysiology of these milder forms of HAND is poorly understood. As the HIV+ population ages, it is becoming increasingly important to distinguish HAND from other forms of dementia that are common in the general population, such as Alzheimer’s disease (AD).

A variety of mechanisms have been proposed to explain HAND pathogenesis in the late HAART era. HIV enters the brain early after acute infection, with macrophages and microglia serving as the major reservoirs of replicating and latent virus. Effective HAART suppresses viral replication but does not affect latent virus, even in maximally treated persons [9]. Interruption of HAART leads to dramatic elevation in peripheral, brain, and CSF viral loads, increasing the risk of serious CNS consequences [10, 11]. In persons on continuous HAART, brief, low-level episodes of viral escape or “blipping” in brain and CSF may contribute to HAND by inducing immune activation and inflammation [12] and dysregulation of the tryptophan-kynurenine pathway, resulting in accumulation of neurotoxic metabolites [13]. The neurovascular unit, consisting of CNS blood vessels and their interface with brain parenchyma, may also be involved in HAND pathogenesis [4]. The neurovascular unit is involved in maintenance of the blood–brain barrier and regulation of microvascular blood flow, so chronic or intermittent disturbance of its components may lead to irreversible damage. Some antiretroviral drugs have been associated with neurotoxic effects, which may also contribute to or exacerbate HAND [14]. Brain function is also influenced by overall health and co-morbidities, including those affecting liver function, such as HCV infection and alcohol use. Therefore, liver disease and other comorbidities are additional factors that could potentially contribute to some forms of HAND [15, 16]. Lastly, some investigators have proposed that HIV infection and/or HAART may cause neurocognitive impairment by disrupting amyloid metabolism [17].

To elucidate mechanisms contributing to HAND pathogenesis in the late HAART era, we examined associations between liver pathology, brain aging, and HAND in studies of brain and liver autopsy tissue from older HIV+ patients on HAART compared to controls during the calendar period 2002–2015 by histopathological methods, a sensitive fluorescent amyloid staining method (AmyloGlo), and targeted gene expression profiling (NanoString).

## Methods

### Cohort selection and characterization

A cohort of older HIV+ patients (defined as > =age 45 at death) and age-matched controls with available autopsy brain frontal lobe and liver tissue was assembled from the National Neurological AIDS Bank (NNAB) at UCLA [18] and Pathology Specimen Locator Core at Brigham and Women’s Hospital (BWH). All subjects were enrolled with written informed consent and Institutional Review Board (IRB) approval at each study site. Autopsy tissue samples, demographic, clinical, and laboratory data were collected and coded to protect participants' confidentiality in accordance with IRB-approved protocols at UCLA, BWH, and DFCI. To identify older HIV- controls, we searched for persons aged 45–80 years with no history of HIV. To identify older HIV+ cases, we searched for participants aged 45–80 on HAART for at least 1 year, including the year prior to death, with a recorded last pre-mortem plasma viral load (VL) within 12 months prior to death of <1000 copies/mL or undetectable (<50 or 400 copies/ml, depending upon the year that the test was obtained). Efforts were made to exclude cases with fatal stroke, CNS hemorrhage, Parkinson’s Disease, multiple sclerosis, active CNS opportunistic infection, neurosyphilis and brain tumor. The following cases not meeting all search or exclusion criteria were included: four cases under age 45 (range, 33–41 years), four cases with last plasma VL >1000 copies/mL (range, 1985–70,953 copies/ml), four cases that stopped HAART in the last few months prior to death, one with hemorrhagic middle cerebral artery stroke, and three with active fungal or bacterial infections involving the CNS at time of death.

### Pathologic evaluation

Formalin-fixed brain frontal lobe and liver tissues were obtained for cases from NNAB. Representative tissue was processed and paraffin-embedded using standard histological protocols by the Specialized Histopathology Services Longwood Core at BWH. Unstained formalin-fixed paraffin-embedded (FFPE) tissue sections were prepared from NNAB and BWH cases. All frontal lobe sections were stained with hematoxylin and eosin (H&E) and luxol fast blue and periodic acid-Schiff (LFB-PAS). Selected cases were also examined by glial fibrillary acidic protein (GFAP) (ab7260, Abcam, Cambridge, UK) and amyloid-beta (Aβ) (M0872, Dako, Glostrup, Denmark) immunohistochemistry. Liver sections were stained with H&E and Masson’s trichrome. All slides were reviewed by two study pathologists.

### AmyloGlo and immunofluorescence staining

FFPE frontal lobe tissue sections were stained with AmyloGlo RTD (Biosensis, Thebarton, Australia) according to the manufacturer’s protocol in phosphate buffered saline for 10 min [19]. Slides were then stained with mouse anti-CD68 monoclonal antibody (M0876, Dako) and rabbit anti-GFAP polyclonal antibody followed by goat anti-mouse AlexaFluor 488 (A-11001 l, Invitrogen, Waltham, MA) and goat anti-rabbit AlexaFluor 647 (A-21245, Invitrogen). Incubation with Sudan Black B (Sigma Aldrich, St. Louis, MO) (1.0% in 70% ethanol) for 10 min was done prior to cover-slipping. Slides were imaged with a Leica SP8 X Confocal Microscope and images analyzed with Fiji [20] and Adobe Photoshop software.

### Gene expression profiling

Subcortical white matter mRNA was extracted from FFPE tissue sections (13 BWH cases), or frozen frontal lobe sections (5 NNAB cases) using a FFPE DNA/RNA isolation kit according to the manufacturer's instructions (Qiagen, Valencia, CA). RNA content and quality (RIN; RNA integrity number) were evaluated using BioAnalyzer (Agilent). mRNA hybridization, detection, and scanning were performed using NanoString nCounter technology and software (NanoString Technologies, Seattle, WA) [21] at the DFCI Molecular Biology core facility. For FFPE samples, RNA input was adjusted to 100 ng based on the percentage of RNA greater than 50–300 bp to normalize for RNA degradation in FFPE tissue samples. Each RNA sample was probed with a custom probe set to detect mRNA transcripts for 24 cellular genes including 11 cell type-specific markers (neuronal, oligodendrocyte, astrocyte, macrophage/microglial, endothelial cells) and 13 inflammation/immune activation markers (see Additional file 1). Eight additional probes to detect housekeeping gene transcripts were used for normalization. Quality control checks and data normalization using internal negative and positive controls and housekeeping genes was performed using nSolver 3.0 software. From 18 cases with RNA isolated from 13 FFPE and five frozen tissue samples, four cases with high expression of neuronal markers (i.e. SNAP25 and SYN1) were excluded from the final analysis, resulting in 14 cases available for analysis of gene expression.

### Statistics

Statistical significance was determined by Student’s *t*-test or Chi-squared test (*p-*values <0.05). For analysis of gene expression using NanoString technology, fold- change and *p*-values were calculated using Student's *t*-test (*p* < 0.05), false discovery rate (FDR) was calculated using fdrtool in R (FDR < 0.10), and semi-supervised heatmaps were generated using R.

## Results

### Cohort characteristics

Nineteen HIV+ and nine HIV- subjects from UCLA were combined with 9 HIV+ and 16 HIV- subjects from BWH, for a total of 28 HIV+ and 25 HIV- subjects examined at autopsy during the late HAART era (2002–2015). Demographic, clinical characteristics, and laboratory data, and differences between the two groups, are summarized in Table 1. The median age of the HIV+ group was approximately 10 years younger compared to the HIV- control group (*p* = 0.001), and included more males (*p* = 0.006), while race showed no significant differences between groups. The median post-mortem interval for HIV- controls was longer compared to HIV+ cases (*p* = 0.03), reflecting the NNAB’s rapid autopsy protocol. HIV+ cases were more likely to be positive for HCV or HBV (*p* = 0.002) and to report use of illicit drugs (mainly cocaine and other stimulants) (*p* <0.0001) or alcohol (*p* = 0.0004). There were no significant differences in BMI, or prevalence of smoking, hypertension, diabetes, hyperlipidemia, or cerebrovascular disease, while cardiovascular disease was slightly more prevalent (*p* = 0.03), and BMI and HgA1c values were higher, in HIV- compared to HIV+ subjects (*p* = 0.04). Cancer was more prevalent in HIV+ cases than controls (*p* = 0.04) and included diagnoses of primary CNS lymphoma, Kaposi sarcoma, squamous cell carcinoma of the tongue, and squamous cell carcinoma of the anus. There were no significant differences in laboratory values related to kidney and liver function or cholesterol homeostasis. Apolipoprotein E (APOE) genotypes were available only for HIV+ cases, of which 3/21 (14.3%) had at least one ε4 allele. Eighty-nine % of HIV+ cases were on HAART, 50% had a last recorded CD4+ count <200 cells/μL (mean 214.7 +/− 161.1), 81.5% had a last recorded plasma VL <400 copies/mL, and 7/8 (87.5%) had undetectable CSF VL (one case had CSF VL 942 copies/mL at time of death). Nine of 28 (32.1%) HIV+ cases had a HAND diagnosis (4 ANI, 2 MND, and 3 HAD) and ten died of AIDS-related causes (e.g., opportunistic fungal infections, *Pneumocystis jirovecii* pneumonia, non-Hodgkin’s lymphoma, Kaposi’s sarcoma).

**Table 1:** Demographics, clinical characteristics, and laboratory data of the study cohort

	HIV-positive (*n* = 28)	HIV-negative (*n* = 25)	*p-*value
Demographics			
Age at death (years)	56.1 (8.9)	64.9 (15.1)	0.001
Gender (male)	23 (82.0)	11 (44.0)	0.006
Race (Caucasian)	23 (42.9)	13 (52.0)	0.5
Post-mortem interval (hours)	13.5 (16.4)	24 (40.5)	0.03
Substance use and comorbidities			
Smoking	16 (57.1)	10 (40.0)	0.2
Illicit drugs	18 (64.3)	0 (0.0)	<0.00001
Alcohol	11 (39.3)	0 (0.0)	0.0004
HCV or HBV infection	15 (46.0)	2 (8.0)	0.002
BMI	22.9 ± 3.8	26.8 ± 6.0	0.05
Hypertension	17 (60.7)	16 (64.0)	0.8
Diabetes	4 (14.3)	5 (20.0)	0.6
Hyperlipidemia	19 (67.9)	17 (68.0)	0.99
Cancer	13 (46.4)	5 (20.0)	0.04
Cardiovascular disease	12 (42.9)	18 (72.0)	0.03
Cerebrovascular disease	12 (42.9)	10 (40.0)	0.99
HAND	9 (32.1)	-	-
Laboratory values			
Creatinine (mg/dL)	2.1 ± 1.7	1.9 ± 1.2	0.7
ALT (U/L)	57.8 ± 63.3	70.5 ± 122.1	0.7
AST (U/L)	80.5 ± 112.0	108.5 ± 140.1	0.5
ALP (U/L)	131.2 ± 201.0	98.1 ± 47.2	0.2
Total bilirubin (mg/dL)	4.4 ± 9.8	1.8 + 4.4	0.3
Total cholesterol (mg/dL)	183.9 ± 79.8	185.5 ± 44.9	0.9
LDL (mg/dL)	78.9 ± 30.5	107.2 ± 43.3	0.09
HDL (mg/dL)	40.3 ± 16.6	49.7 ± 16.1	0.2
Triglycerides (mg/dL)	172.7 ± 62.3	170.6 ± 86.8	0.9
HgA1c (%)	5.1 ± 0.7	6.3 ± 1.6	0.04
APOE genotype (E4)	3 (14.3)	ND	-
HIV-related characteristics			
CD4 count (cells/μL)	214.7 ± 161.1	-	-
CD4 count <200 cells/μL	14 (50.0)		
Plasma viral load (copies/mL)	295 (1307)	-	-
Plasma viral load <400 copies/mL	22 (81.5)		
CSF viral load <40 copies/mL	7 (87.5)	-	-
On HAART	25 (89.3)	-	-
AIDS-related causes of death	10 (35.7)	-	-

Values represent n (%), mean ± STDEV, or median (IQR). *p*-values from Student’s *t*-test or Chi-squared test

*Abbreviations*: *HCV* hepatitis C virus, *HBV* hepatitis B virus, *BMI* body mass index, *HAND* HIV-associated neurocognitive disorders, *ALT* alanine transaminase, *AST* aspartate transaminase, *LDL* low-density lipoprotein, *HDL* high-density lipoprotein, *HgA1c* hemoglobin A1C, *APOE* apolipoprotein E, *CSF* cerebrospinal fluid; HAART, highly-active antiretroviral therapy, *ND* not done. Cerebrovascular disease is based on medical records and autopsy findings. HAND diagnoses include: four asymptomatic neurocognitive impairment (ANI), two minor neurocognitive disorder (MND), and three HIV-associated dementia (HAD). Laboratory values are the last recorded pre-mortem values within 12 months prior to death

### Brain histopathologic features

**Fig. 1:**
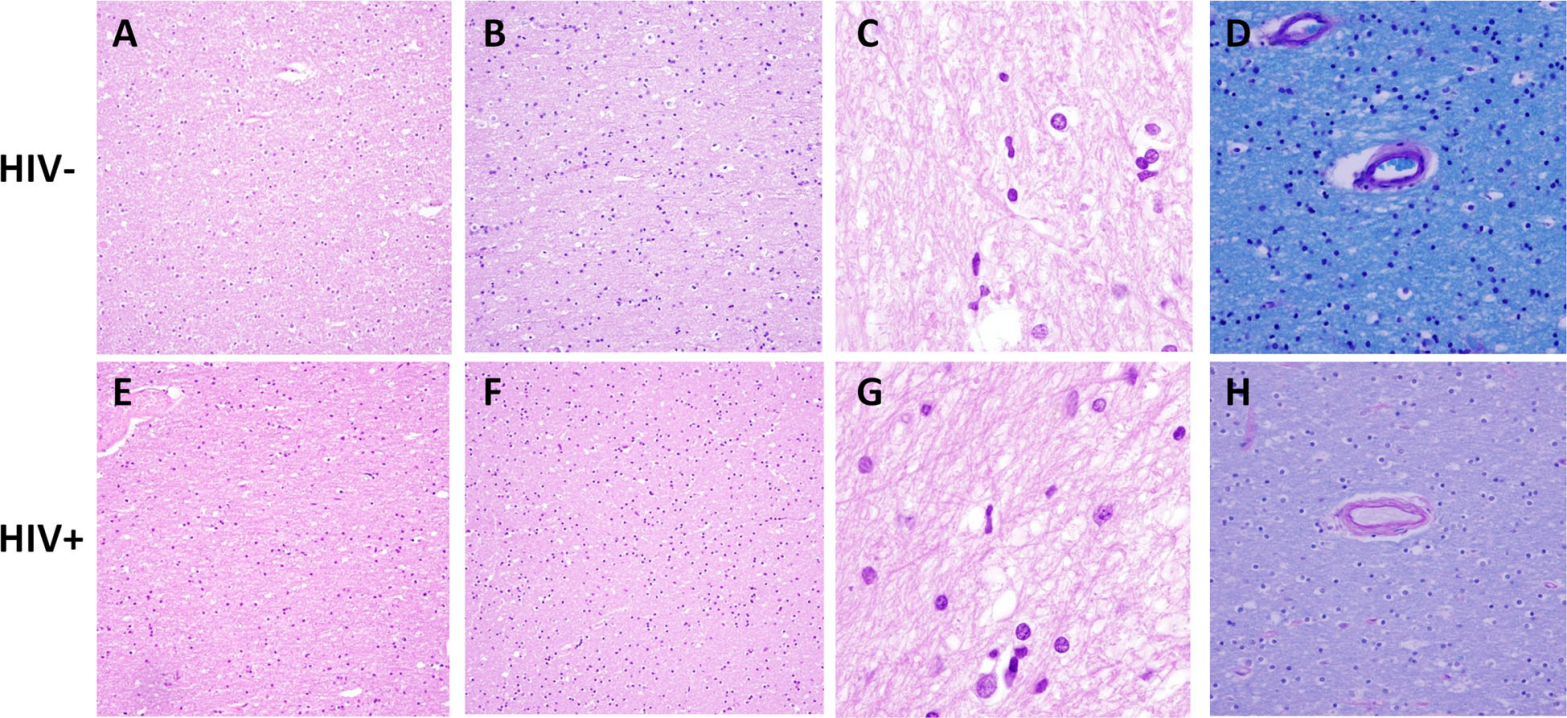
Brain frontal lobe histopathologic findings in HIV+ cases and controls. Representative formalin-fixed paraffin embedded autopsy brain frontal lobe tissue from HIV- (**a**-**d**) and HIV+ (**e**-**h**) cases stained with hematoxylin and eosin (**a**-**c**, **e**-**g**) or luxol fast *blue* and periodic acid-Schiff (**d**, **h**). A minority of HIV+ and HIV- cases were histologically unremarkable (**a**, **e**). Many HIV+ and HIV- cases exhibited mild histopathologic abnormalities including hypercellular white matter (**b**, **f**), increased rod cells (microglia) (**c**, **g**), and/or moderately thickened blood vessels (**d**, **h**). Original magnification 200× (**a**-**b**, **e**-**f**), 400× (**d**, **h**), and 1000× (**c**, **g**)

**Fig. 2:**
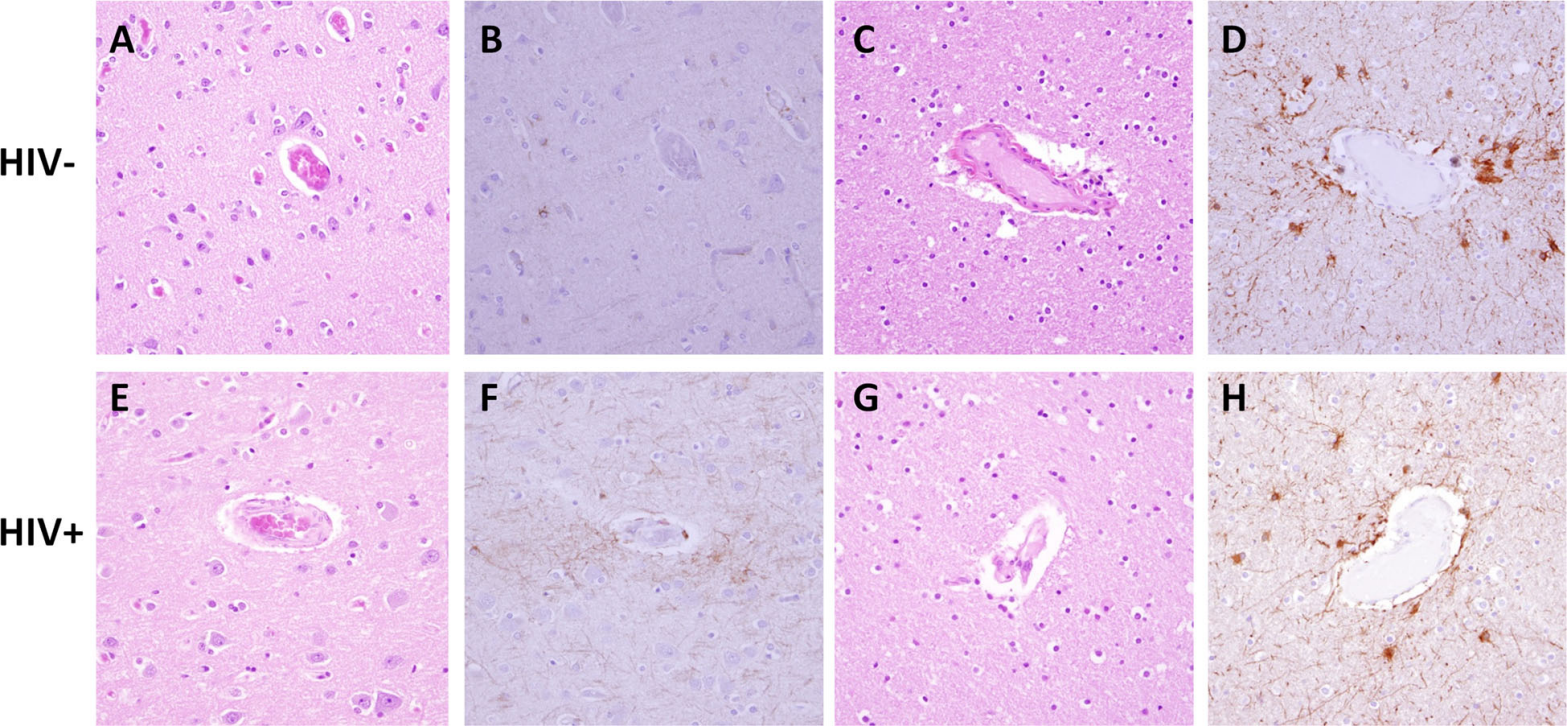
*Grey* and *white* matter gliosis in HIV+ cases compared to controls. Representative formalin-fixed paraffin embedded autopsy brain frontal lobe tissue from HIV- (**a**-**d**) and HIV+ (**e**-**h**) cases stained with hematoxylin and eosin (**a**, **c**, **e**, **g**) or glial fibrillary acidic protein (GFAP) immunohistochemistry. *Grey* matter exhibited minimal gliosis (**a**, **b**, **e**, **f**), while the majority of HIV+ and HIV- cases demonstrated diffuse *white* matter gliosis, and a minority of cases exhibited focal perivascular gliosis (**c**, **d**, **g**, **h**). Original magnification 400×

In autopsy reports, the most common brain pathologies were infarcts/ischemia (45.3%), hemorrhage/hematoma (9.4%), large vessel atherosclerosis (7.5%), arterial/arteriolar sclerosis (11.3%), and evidence of current or prior infection (e.g. cryptococcal meningitis, aspergillus) (17.0%). FFPE brain frontal lobe tissue, including grey matter and subcortical white matter, was available for evaluation in 27 HIV+ and 22 HIV- cases, the results of which are summarized in Table 2. A minority of HIV+ and HIV- cases (11.1% and 9.1%, respectively) were histopathologically unremarkable (Fig. 1a, e). Many HIV+ and HIV- cases exhibited mild histopathologic abnormalities including hypercellular white matter (Fig. 1b, f), increased rod cells (microglia) (Fig. 1c, g), moderately thickened blood vessels (highlighted by PAS stain) (Fig. 1d, h), or myelin loss (demonstrated by LFB stain), but there were no statistically significant differences between the groups. Grey matter exhibited minimal gliosis (Fig. 2a-b, e-f), while the majority of HIV+ and HIV- cases demonstrated diffuse white matter gliosis by GFAP immunohistochemistry, and a minority of cases exhibited focal perivascular gliosis (Fig. 2c-d, g-h). A non-statistically significant trend (*p* = 0.09) suggested a greater proportion of cases with gliosis in the HIV+ group compared to controls. Inflammation was present in a minority of cases including five HIV+ cases with chronic inflammatory infiltrates compared to three controls with chronic and one control with acute inflammatory infiltrates. Brain histopathologic features listed in Table 2 showed no significant associations with HAND (all *p*-values > 0.10, chi-squared test; *n* = 9 with HAND vs. *n* = 18 without HAND).

**Table 2:** Brain frontal lobe histopathologic features

Histologic feature	Assessment	HIV-positive (*n* = 27)^a^	HIV-negative (*n* = 22)^a^	*p*-value
		n (%)	n (%)	
Astrocytes	Normal	16 (59.3)	18 (81.8)	0.09
(H&E)	Increased	11 (40.7)	4 (18.2)	
Activated astrocytes	Diffuse white matter	17 (100)	15 (93.8)	0.3
(GFAP IHC)	White matter vessels	3 (17.6)	1 (6.25)	0.3
	Grey matter	4 (23.5)	3 (18.8)	0.7
Microglia	Normal	9 (33.3)	7 (31.8)	0.9
	Increased	18 (66.7)	15 (68.2)	
Myelin	Normal	15 (55.6)	13 (59.1)	0.8
	Loss	12 (44.4)	9 (40.9)	
Blood vessel thickening	None	19 (70.4)	13 (59.1)	0.6
	Mild	4 (14.8)	3 (13.6)	
	Moderate/severe	4 (14.8)	6 (27.3)	
Inflammation	None	19 (70.4)	17 (77.3)	0.5
	Chronic	5 (18.5)	3 (13.6)	
	Acute	0 (0.0)	1 (4.5)	
Amyloid deposition				
(AmyloGlo)	positive	1 (5.9)	2 (12.5)	0.5

Values listed represent n (%). *p*-values from Chi-squared test

*Abbreviations*: *GFAP* glial fibrillary acidic protein, *IHC* immunohistochemistry

^a^For GFAP IHC and AmyloGlo, *N* = 17 for HIV+ and *N* = 16 for HIV-

### Amyloid deposition in brain

**Fig. 3:**
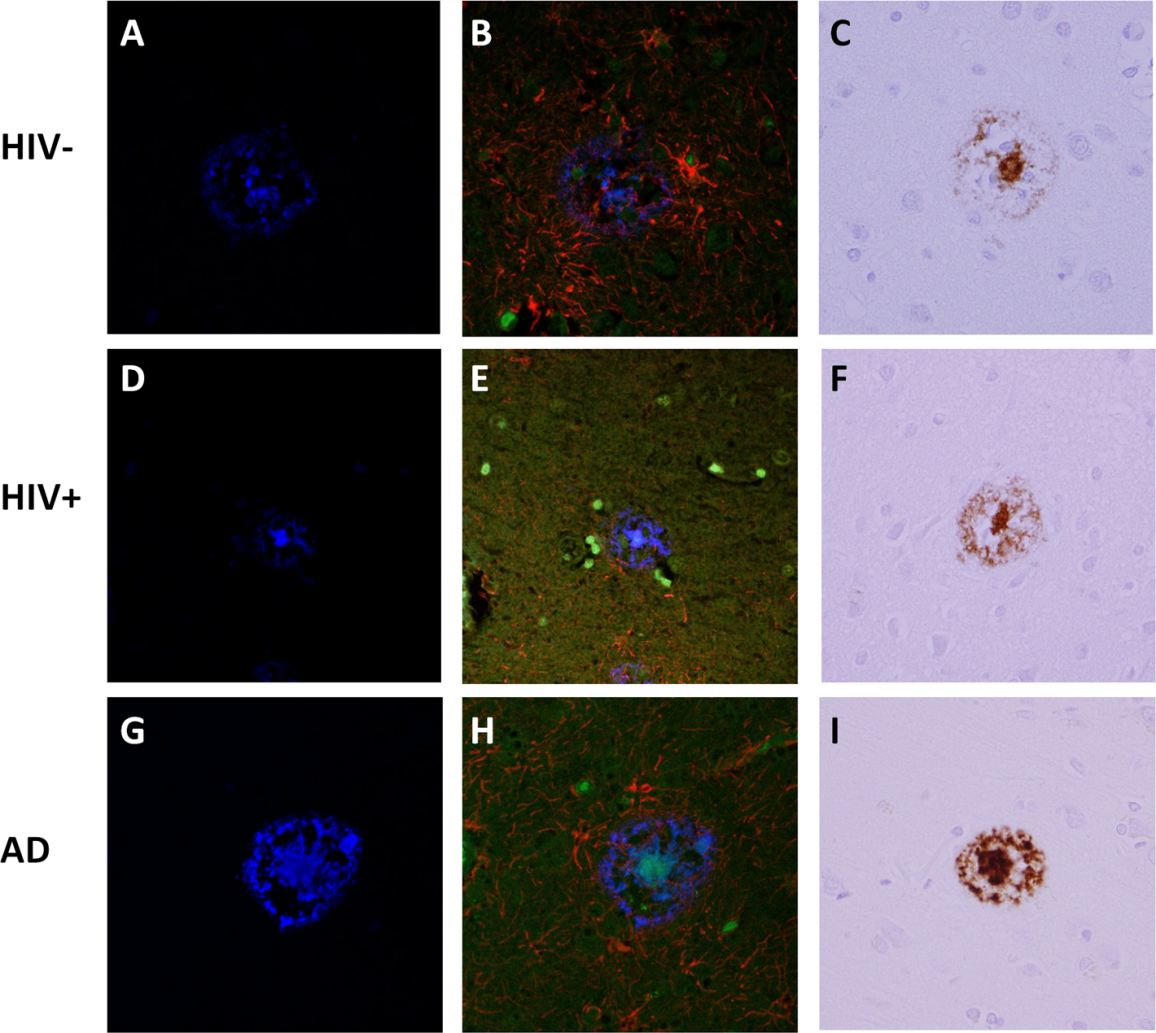
Fluorescent staining of brain amyloid in HIV+ cases and controls. Representative formalin-fixed paraffin embedded autopsy brain frontal lobe tissue from HIV- (**a**-**c**) and HIV+ (**d**-**f**) cases and a known Alzheimer’s disease (AD) case (**g**-**i**) were examined by AmyloGlo staining (*blue*) (**a**-**b**, **d**-**e**, **g**-**h**), CD68 (*green*) and GFAP (*red*) immunofluorescence (**b**, **e**, **h**), or amyloid-beta (Aβ) immunohistochemistry (**c**, **f**, **i**). Large amyloid plaques with dense central cores were identified in one HIV+ and two HIV- cases, with peripheral GFAP-positive astrocytes and rare CD68-positive microglia. The plaques were confirmed to contain Aβ protein, and exhibited similar morphology to the positive control (AD) case. Original magnification 400×

Staining for amyloid deposits with the fluorescent compound AmyloGlo was performed on FFPE frontal lobe sections for 17 HIV+ and 16 HIV- cases, with GFAP and CD68 immunofluorescent co-staining. Large amyloid plaques with dense central cores were identified in only one HIV+ (69-year-old female with Alzheimer’s disease histopathology at autopsy) and two HIV- cases (66 year old male and 74 year old female) (Fig. 3a-b, d-f). All three cases had GFAP-positive astrocytes adjacent to amyloid plaques, and rare CD68-positive microglia. Morphology of the amyloid plaques was similar to the positive control case (Braak Stage V-VI Alzheimer’s disease), as demonstrated by Aβ immunohistochemistry (Fig. 3c, f, i).

### Liver histopathologic findings

**Fig. 4:**
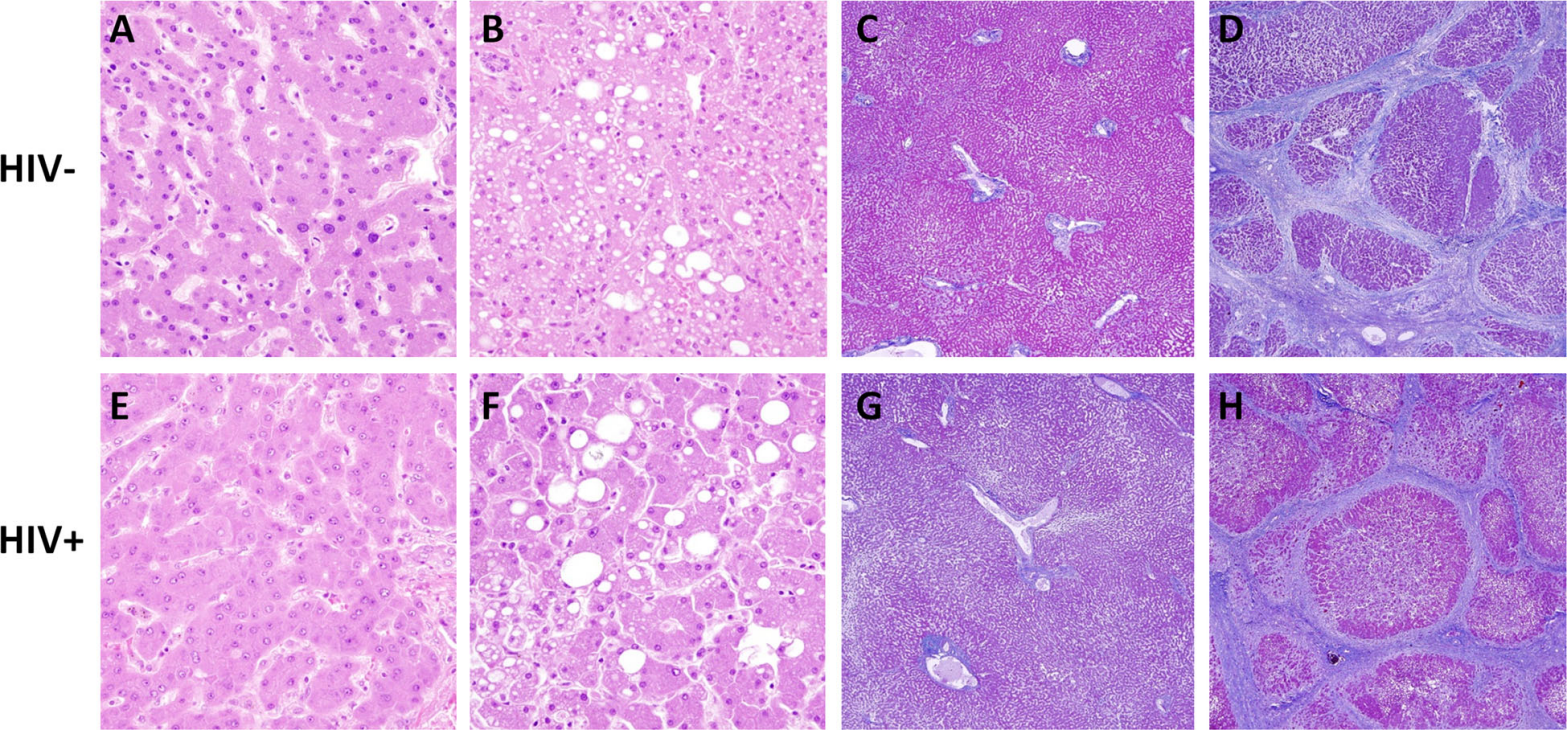
Liver histopathologic findings in HIV+ cases and controls. Representative formalin-fixed paraffin embedded autopsy liver tissue from HIV- (**a**-**d**) and HIV+ (**e**-**h**) cases stained with hematoxylin and eosin (**a**-**b**, **e**-**f**) or Masson’s trichrome stain (**c**-**d**, **g**-**h**). A minority of HIV+ and HIV- cases were histologically unremarkable (**a**, **c**, **e**, **g**), while several HIV+ and HIV- cases exhibited moderate to severe micro- and macrovesicular steatosis (**b**, **f**) and/or severe fibrosis/cirrhosis (**d**, **h**). Original magnification 40× (**c**-**d**, **g**-**h**) or 400× (**a**-**b**, **e**-**f**)

In autopsy reports, the most common liver pathologies were micro- or macro-steatosis (26.4%), severe fibrosis/cirrhosis (24.5%), inflammatory infiltrates (24.5%), necrosis (13.2%), and primary or metastatic carcinoma (9.4%). FFPE liver was available for evaluation in 24 HIV+ and 18 HIV- cases, with results summarized in Table 3. A minority of HIV+ and HIV- cases (16.7% and 16.7%, respectively) were histologically unremarkable (Fig. 4a, e). Moderate to severe micro-and macrovesicular steatosis was more common in controls (66.7%) than in HIV+ cases (41.6%, *p* = 0.03) (Fig. 4b, f), while severe fibrosis/cirrhosis was more common in HIV+ cases (58.3%) than in controls (33.3%, *p* = 0.02) (Fig. 4d, h). Cases with mild or moderate to severe inflammation and necrosis were identified in both HIV+ and control groups without significant differences in prevalence between groups. Bile duct proliferation (*p* = 0.03) and lipofuscin (*p* = 0.04) but not bile pigment deposition were more prevalent in HIV+ cases than controls. In 39 cases with available paired liver and brain sections, there were no significant associations between moderate to severe liver pathology and brain pathologies listed in Table 2 (all *p*-values ≥ 0.10; *n* = 14 no/mild liver pathology vs. *n* = 25 moderate/severe liver pathology, chi-squared test of groups stratified by HIV status). We also found no significant associations between moderate to severe liver pathology and HAND (*p* = 0.11, chi-squared test; *n* = 9 with HAND vs. *n* = 18 without HAND).

**Table 3:** Liver histopathologic features

Histologic feature	Assessment	HIV-positive (*n* = 24)	HIV-negative (*n* = 18)	*p*-value
		n (%)	n (%)	
Fibrosis (grade)	1	3 (12.5)	9 (50.0)	0.02
	2	7 (29.2)	3 (16.7)	
	3	3 (12.5)	4 (22.2)	
	4 - cirrhosis	11 (45.8)	2 (11.1)	
Inflammation	None	9 (37.5)	6 (33.3)	0.6
	Mild	6 (25.0)	7 (38.9)	
	Moderate/severe	6 (25.0)	4 (22.2)	
	Necrosis	3 (12.5)	1 (5.6)	
Steatosis	None	10 (41.7)	4 (22.2)	0.03
	Focal	4 (16.7)	2 (11.1)	
	Moderate	2 (8.3)	9 (50.0)	
	Severe	8 (33.3)	3 (16.7)	
Bile duct proliferation	Present	10 (41.7)	2 (11.1)	0.03
Pigments	Bile	11 (45.8)	3 (16.7)	0.05
	Lipofuscin	7 (29.2)	11 (61.1)	0.04

Values listed represent n (%). *p*-values from Chi-squared test

### Gene-expression profiles

**Fig. 5:**
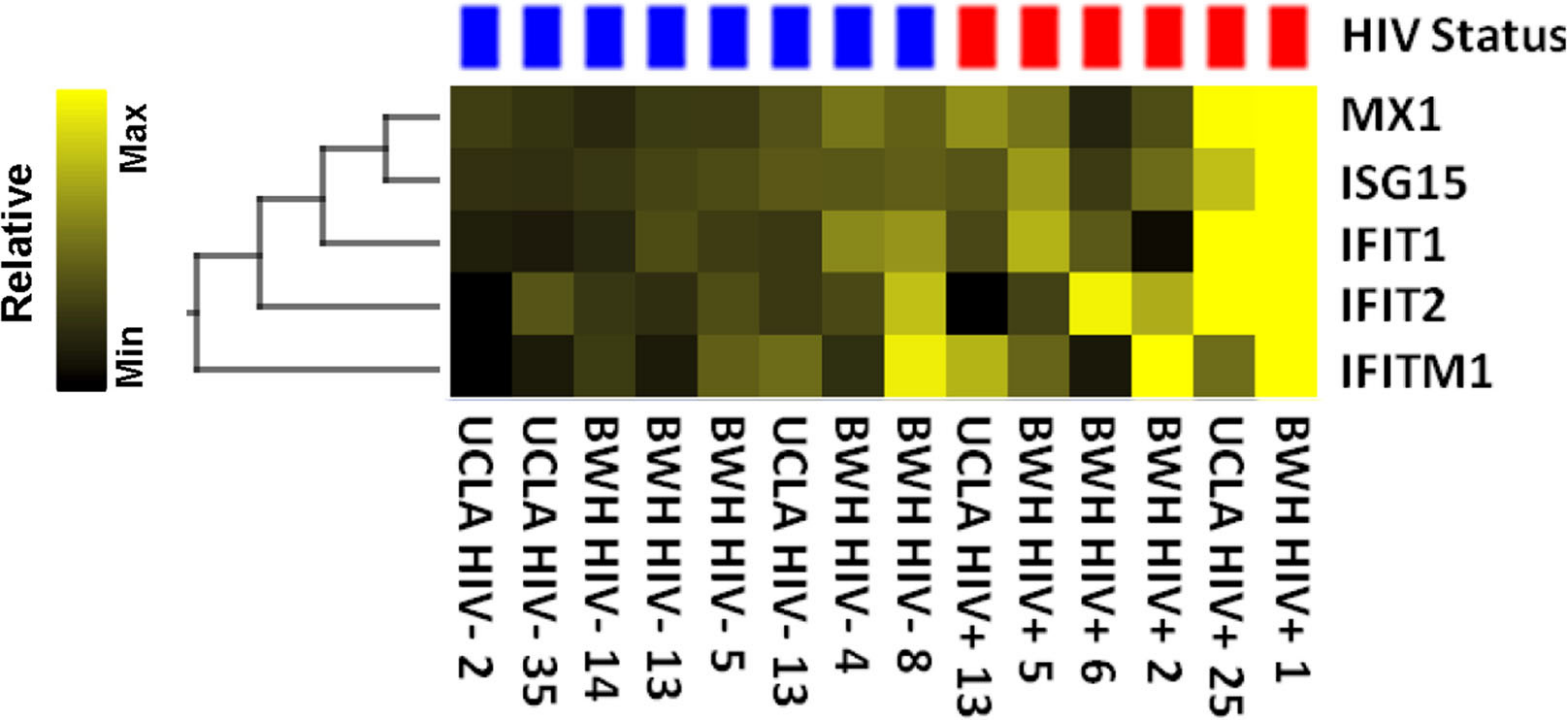
Expression of interferon-response genes in frontal lobe subcortical white matter from HIV+ cases compared to controls. Semi-supervised heatmap showing increased expression and hierarchical clustering of interferon-response genes in white matter from HIV+ cases compared to controls. One hundred nanograms of RNA from HIV+ and HIV- cases was probed to detect mRNA transcripts using the NanoString nCounter platform (see Additional file 1). *Red* and *blue boxes* indicate HIV+ and HIV- cases, respectively

Subcortical white matter from 6 HIV+ and 12 HIV- cases was isolated and mRNA extracted for gene expression profiling using the NanoString nCounter platform to detect a targeted panel of 24 genes, consisting of genes involved in interferon responses and inflammation, and cell-type specific markers of macrophages/microglia, astrocytes, oligodendrocytes, neurons, and endothelial cells. After excluding four samples with high expression of neuronal gene transcripts (i.e. SNAP25 and SYN1), indicating significant contamination with grey matter, six HIV+ and eight HIV- cases were available for analysis. After normalization of values using eight housekeeping genes to control for variability in quantity and quality of input RNA, 24 cellular genes were examined for differences in mRNA transcript levels between HIV+ cases vs. controls (see Additional file 1). A set of five genes involved in interferon (IFN) responses, MX1, ISG15, IFIT1, IFIT2, and IFITM1, showed a trend towards higher expression of mRNA transcripts in HIV+ cases compared to controls (*p* = 0.06; Fig. 5 and Additional file 1). This difference was largely driven by two HIV+ cases with high levels of IFN-response gene transcripts (BWH HIV+ 1 and UCLA HIV+ 25 with autopsy diagnoses of sepsis with endocarditis and microemboli in brain and other tissues, and with Alzheimer’s disease, respectively); the remaining four HIV+ cases had minimal to moderate elevations in IFN-induced gene transcripts compared to controls. To exclude the possibility that differences in RNA quality accounted for differences in levels of IFN response gene transcripts, we examined RIN values, which ranged from 2.1 to 4.7 (mean 2.6 +/−0.73); these low values were expected given that RNA was isolated from autopsy FFPE tissues. RIN values showed no association with differences in levels of IFN-response mRNA transcripts, indicating that differences in levels of IFN-response gene expression were not attributable to differences in the quantity or quality of input RNA.

## Discussion

This is the first comparative study of brain and liver histopathology in older HIV+ patients on ART with low or undetectable VL in the late HAART era. As expected, classic findings of HIVE, such as multinucleated giant cells and microglial nodules, were not observed. Instead, we observed nonspecific histologic findings commonly seen in autopsy brain from HIV+ patients in the late HAART era, such as mild histopathologic abnormalities, anoxic-ischemic changes, focal parenchymal and subarachnoid hemorrhage, Alzheimer type II astrocytes (indicative of hepatic encephalopathy), and primary CNS or systemic lymphoma [7, 22, 23]. Contrary to expectations, frontal lobe pathology of HIV+ and HIV- cases was surprisingly similar, and showed no significant associations with liver pathology or HAND. Deep white matter from HIV+ cases showed increased gliosis compared to HIV- controls, but no differences in myelin loss, blood vessel thickening, or inflammation. With regard to liver pathology, severe fibrosis/cirrhosis was more common in HIV+ cases, largely attributable to HCV or HBV co-infection, while moderate to severe steatosis was more common in controls and frequency of liver inflammation and necrosis was similar between groups. HIV+ subjects were reasonably well matched to controls for many clinical characteristics including smoking and vascular risk factors, with exception of the younger median age (56 vs. 65 years in HIV+ and HIV-, respectively). Moreover, the results we report remained similar using data from HIV+ vs. HIV- groups more closely matched for median age (*n* = 24 HIV+ vs. *n* = 20 HIV-; median age 57 vs. 62 years, *p* = 0.133) or from the HIV+ cases with controlled VL (<400 HIV RNA copies/mL) and no evidence of active infections near time of death (*n* = 21 HIV+ vs. *n* = 25 HIV-).

Accelerated aging has been proposed to occur in HIV patients and contribute to mechanisms underlying HAND among older HIV+ individuals [6, 24, 25]. Previous studies reported that levels of Aβ deposited in brain correlate with brain HIV viral loads [23]. However, other studies found no correlation between cognitive deficits and Aβ levels [26, 27], so there is lack of consensus regarding Aβ deposits in HAND. To assess the burden of Aβ deposits in frontal lobe, we used AmyloGlo, a fluorescent amyloid stain with advantages over alternative compounds due to its excitability in the UV range, and suitability for multiple immunofluorescence labeling [19]. Large, predominantly cortical amyloid deposits with dense cores were detected in only one HIV+ patient, a 69-year-old black female with unsuppressed CSF VL (942 copies/mL) and AD pathology at time of autopsy; amyloid plaques were surrounded by GFAP-positive reactive astrocytes and scattered CD68-positive microglia. No definitive clinical diagnosis of HAND or AD was made for this subject, who had additional co-morbidities prior to death, including cocaine use, myocardial infarction, and metastatic gastric carcinoma. Two control cases (ages 66 and 74 years) without clinical diagnoses of AD also had Aβ deposition in brain detected by Amyloglo. Although several studies reported Aβ plaques in HIV+ individuals with predominance of diffuse plaques or intraneuronal deposits [28–30], we detected no morphologic differences between HIV+ and HIV- Aβ deposits. Some studies reported increased rates of HAND with Aβ plaques among APO ε4 carriers, while others reported no increased risk [30–33]. In our study, no amyloid plaques were identified in two HIV+ cases with APO ε4 genotype, and the HIV+ case with amyloid plaques was an APO ε3 homozygote. Published reports of HIV+ patients with cognitive features of AD are rare, in part due to difficulties distinguishing AD from HAND and other comorbidities [34, 35]. Taken together, these findings argue against accelerated amyloid deposition among HIV+ individuals.

Cerebrovascular disease causes significant morbidity in the HIV+ population, where increased prevalence in younger individuals has been associated with viral replication and inflammation, as well as dyslipidemia and pro-atherogenic effects of some ART drugs [6, 36]. HIV infection is an independent risk factor for stroke, and untreated cerebrovascular risk factors, including hypertension and hyperlipidemia, have detrimental effects on neurocognition [37–40]. Chronic inflammation associated with HIV infection has been implicated in promoting atherosclerosis, and an increase in brain large artery inflammation was recently demonstrated in HIV+ patients [41]. Compared to several prior studies, HIV+ subjects in our cohort were of similar age or slightly older, with higher prevalence of cerebrovascular risk factors including smoking, illicit drug use, hypertension, hyperlipidemia, and diabetes. Some of these cohort differences may reflect the population recruited by NNAB, which preferentially recruits individuals with severe illness likely to result in mortality, compared to other cohorts. In contrast to many prior studies, cardiovascular disease was more prevalent and HgA1c was higher in the HIV- group in our study cohort compared to controls. The HIV- group in our study was ~10 years older than the HIV+ group, raising the possibility that the lack of a significant difference in prevalence of cerebrovascular disease between groups may also reflect the younger age of the HIV+ subjects. Supporting this hypothesis, the ages of HIV+ cases with moderate to severe cerebrovascular pathology ranged from 39 to 68 years (median 58), while the ages of HIV- controls with similar pathology ranged from 54 to 77 years (median 73).

Liver disease including chronic viral hepatitis, drug-associated hepatotoxicity, non-alcoholic fatty-liver disease, and opportunistic infections accounts for significant morbidity and mortality in the HIV+ population [42, 43]. Failure of the liver to remove toxins from the blood causes hepatic encephalopathy via mechanisms involving increased ammonia and inflammation, and is histologically associated with the presence of Alzheimer type II astrocytes in brain [44]. HIV co-infection with HCV or HBV occurs frequently, due to shared routes of transmission, often progressing to cirrhosis. In some studies, liver fibrosis has been associated with adverse effects on cognitive performance in HIV/HCV co-infected individuals [45]. The liver plays a central role in cholesterol metabolism and clotting, which can have profound systemic and CNS effects [46]. Previous histopathologic studies reported frequent fatty changes, inflammation, cirrhosis, bile stasis, and carcinoma in livers of HIV+ patients at autopsy [22, 47]. Similar findings were identified in the present study, including, fibrosis/cirrhosis, steatosis, inflammation, and pigment deposition. Severe fibrosis/cirrhosis was more common in HIV+ individuals than controls, which can be attributed to significantly higher rates of HCV co-infection and alcohol consumption. Micro- and macrosteatosis were more common in the HIV- subjects, and there was no significant difference in inflammation, blood vessel thickness, or pigment deposition. Alzheimer type II astrocytes were observed in frontal subcortical white matter in three HIV+ cases, all with HCV co-infection. Despite the frequency of these findings, no consistent histologic patterns were identified in frontal lobe or liver from HIV+ patients and we found no significant associations between liver and brain pathologies.

To elucidate molecular mechanisms contributing to HAND, gene expression profiling was performed on frontal subcortical white matter using the NanoString nCounter platform with a targeted panel of cell-type specific and inflammatory markers. To our knowledge, this is the first study to utilize NanoString technology to study HIV neuropathogenesis in FFPE tissue. NanoString is a useful technology for the study of FFPE tissues, which are generally more available than frozen tissue. A significant advantage is the ability to obtain quantitative signals from low RNA concentrations, or partially degraded RNA, due to signal detection by short probes that do not require an amplification step. Customization of the probe set allowed for inclusion of neuronal markers, which we used to identify and exclude samples with significant grey matter contamination from the final analysis of white matter samples. Prior studies using the Affymetrix array platform examined gene expression in white matter from HIV+ individuals with and without inflammation and cognitive deficits [48–50]. These studies demonstrated elevated expression of IFN-response genes in patients with HIVE and modest elevations in expression of these genes in HIV-infected patients without HIVE or neurocognitive impairment. Consistent with these studies, we detected a trend towards increased expression of the IFN-response genes MX1, ISG15, IFIT1, IFIT2, and IFITM1 in HIV+ cases compared to controls. RIN values were low, as expected for FFPE tissue, but showed no association with IFN-response transcript levels, arguing against low RNA quality or longer PMI as the explanation for differences in gene expression levels. Two HIV+ cases had particularly high expression of these genes, both black females (ages 58 and 69) with histories of illicit drug use, last CD4 cell counts < 300 cells/μL, on HAART with last plasma VL < 400 copies/mL: one had endocarditis and sepsis with microemboli in multiple organs including brain, while the other had Alzheimer’s disease pathology and positive AmyloGlo staining. Given these comorbidities, increased IFN-response gene expression in these two cases was not clearly attributable to HAND. The remaining four HIV+ cases had only minimal to moderate elevations of IFN-induced gene expression compared to controls. Histologic evidence of chronic inflammation was present in 2/6 (33%) HIV+ cases (including the 58-year-old female with endocarditis) compared to 1/8 (12.5%) HIV- cases. Inflammatory infiltrates and IFN-responses in brain are often localized in a patchy distribution, and might be detected in more cases if additional areas were sampled. While these gene expression data have limitations and show only a trend toward statistical significance, they are in agreement with previous studies [48, 50–52]. Thus, IFN-induced gene expression may represent a core signature indicative of ongoing immune activation mediated through IFN response pathways even in HIV+ individuals with suppressed VL on ART in the late HAART era.

While this study has many strengths, there were several limitations including inherent bias related to selection of autopsy subjects, quality of post-mortem tissues available, and availability of clinical and laboratory data. The majority of HIV+ cases were from the NNAB at UCLA, which preferentially recruits HIV+ individuals at risk of death who agree to contribute their tissues for research, increases the likelihood of identifying subjects with neurocognitive deficits. Conversely, the majority of HIV- cases came from routine hospital autopsies at BWH, which includes many patients suffering from chronic diseases, cardiovascular disease, prolonged care in the intensive care unit, and/or unexplained causes of death. BWH cases had longer PMIs than NNAB cases, as NNAB stresses rapid autopsy whenever possible. However, tissue from BWH was more rapidly processed and paraffin-embedded than tissue from NNAB. Prolonged formalin-fixation precluded recovery of usable mRNA for gene expression studies from NNAB cases, requiring analysis of RNA isolated from frozen tissue available for a subset of cases. Clinical and laboratory data was not complete for all patients, including lack of formal evaluation for HAND in BWH HIV+ cases, likely underestimating some co-morbidities. Lastly, the sample size was relatively small, which limited statistical power for some analyses, particularly for gene expression profiling. Larger, prospective studies with focused data collection from HIV+ subjects with controlled VL and no other active infections near time of death are needed to avoid these issues in future studies and gain new insights into pathological mechanisms in successfully treated patients.

## Conclusions

This study is the first to compare paired brain and liver histopathology in older HIV+ individuals with suppressed viral loads in the late HAART era with HIV seronegative controls. Several lines of evidence suggest connections between liver function and brain pathology in the general population due to the role of the liver in detoxifying blood, cholesterol metabolism, and clotting, and modulation of systemic inflammation and vascular disease. Although significant histopathologic findings were identified in both frontal lobe and liver specimens, no consistent patterns or inter-relationships between liver and frontal lobe histopathology were identified in HIV+ individuals. AmyloGlo was used for the first time in this setting, and may prove useful for future studies of Alzheimer’s disease and brain aging in HIV+ individuals. Gene expression profiling using NanoString revealed a trend towards increased IFN response genes, in agreement with prior studies, implicating chronic inflammation as one of several possible factors contributing to the development of neurocognitive deficits in this population. Examination of a larger number of samples with a broader panel of targets as well as new molecular pathology tools are needed to elucidate mechanisms underlying mild forms of HAND in the era of newer HAART regimens, and define the relationship of HAND to comorbidities involving other organ systems.

## Additional file

Additional file 1: Gene expression profiling of frontal lobe subcortical white matter. Table listing 24 genes profiled using the Nanostring nCounter platform to quantify mRNA transcripts. (DOCX 13 kb)

## Abbreviations

AD: Alzheimer’s disease
ANI: Asymptomatic neurocognitive impairment
APOE: Apolipoprotein E
ART: Antiretroviral therapy
Aβ: Amyloid beta
BMI: Body mass index
BWH: Brigham and Women's Hospital
FFPE: Formalin-fixed paraffin-embedded
GFAP: Glial fibrillary acidic protein
HAART: Highly active antiretroviral therapy
HAD: HIV-associated dementia
HAND: HIV-associated neurocognitive disorder
HBV: Hepatitis B virus
HCV: Hepatitis C virus
HIV: Human immunodeficiency virus
IFN: Interferon
LFB-PAS: Luxol fast blue and periodic acid-Schiff
MND: Minor neurocognitive disorder
NNAB: National Neuro AIDS Bank
PMI: Post mortem interval
UCLA: University of California Los Angeles
VL: Viral load
