# Supplementary material for: Brain and liver pathology, amyloid deposition, and interferon responses among older HIV-positive patients in the late HAART era
Source: BMC Infect Dis. 2017 Feb 17;17:151. doi: 10.1186/s12879-017-2246-7 (PMC5316187; doi:10.1186/s12879-017-2246-7)
Supplement: Additional file 1: — Gene expression profiling of frontal lobe subcortical white matter. Table listing 24 genes profiled using the Nanostring nCounter platform to quantify mRNA transcripts. (DOCX 13 kb) [file 12879_2017_2246_MOESM1_ESM.docx]

| **Additional File 1. Gene expression profiling of frontal lobe subcortical white matter** | | | | |
| --- | --- | --- | --- | --- |
| **Gene Name** | **Classification** | **Fold Change** | ***p*-value** | **FDR** |
| MAG | Oligodendrocytes | 1.64 | 0.1991 | 0.07685 |
| MBP | Oligodendrocytes | -1.45 | 0.2387 | 0.07989 |
| OLIG1 | Oligodendrocytes | 1.23 | 0.3726 | 0.09094 |
| SNAP25 | Neurons | 1.66 | 0.5479 | 0.10990 |
| SYN1 | Neurons | 1.46 | 0.5941 | 0.11382 |
| GRIN1 | Neurons | 1.29 | 0.7220 | 0.12304 |
| GRIN2A | Neurons | 1.35 | 0.6863 | 0.12068 |
| GFAP | Astrocytes/glial responses | -1.16 | 0.6589 | 0.11876 |
| CD14 | Macrophages/microglia | 1.75 | 0.3216 | 0.08647 |
| CD68 | Macrophages/microglia | 1.58 | 0.5386 | 0.10906 |
| PECAM1 | Endothelial cells | 1.67 | 0.2720 | 0.08188 |
| IRF1 | IFN response | 1.35 | 0.3369 | 0.08771 |
| IRF3 | IFN response | 1.49 | 0.0940 | 0.06118 |
| IRF7 | IFN response | 1.63 | 0.2132 | 0.07803 |
| **IFIT1** | **IFN response** | **1.95** | **0.0794** | **0.06118** |
| **IFIT2** | **IFN response** | **1.67** | **0.0490** | **0.06118** |
| **IFITM1** | **IFN response** | **2.09** | **0.0746** | **0.06118** |
| **ISG15** | **IFN response** | **3.67** | **0.0810** | **0.06118** |
| **MX1** | **IFN response** | **2.55** | **0.0806** | **0.06118** |
| CXCL9 | Cytokines/chemokines | 6.79 | 0.1457 | 0.07090 |
| CXCL10 | Cytokines/chemokines | 11.37 | 0.1498 | 0.07146 |
| IL1B | Cytokines/chemokines | -1.29 | 0.6331 | 0.11687 |
| IL6 | Cytokines/chemokines | -1.10 | 0.8529 | 0.14218 |
| TNF | Cytokines/chemokines | -2.90 | 0.2727 | 0.08192 |
| Fold change values were calculated following normalization of raw data values using housekeeping genes. *p*-values from Student’s t-test, FDR values from fdr.tool. Bold entries indicate genes included in Figure 5. Abbreviations: FDR, false discovery rate. | | | | |
